# Tumor Necrosis Factor - Alpha Is Essential for Angiotensin II-Induced Ventricular Remodeling: Role for Oxidative Stress

**DOI:** 10.1371/journal.pone.0138372

**Published:** 2015-09-17

**Authors:** Srinivas Sriramula, Joseph Francis

**Affiliations:** 1 Comparative Biomedical Sciences, School of Veterinary Medicine, Louisiana State University, Baton Rouge, Louisiana, United States of America; 2 Department of Pharmacology and Experimental Therapeutics, Louisiana State University Health Sciences Center, New Orleans, Louisiana, United States of America; Cedars Sinai Medical Center, UNITED STATES

## Abstract

The functional crosstalk between angiotensin II (Ang II) and tumor necrosis factor (TNF)-α has been shown to cause adverse left ventricular remodeling and hypertrophy in hypertension. Previous studies from our lab showed that mice lacking TNF-α (TNF-α^-/-^) have attenuated hypertensive response to Ang II; however, the signaling mechanisms involved are not known. In this study, we investigated the signaling pathways involved in the Ang II and TNF-α interaction. Chronic Ang II infusion (1μg/kg/min, 14 days) significantly increased cardiac collagen I, collagen III, CTGF and TGF-β mRNA and protein expression in wild-type (WT) mice, whereas these changes were decreased in TNF-α^-/-^ mice. TNF-α^-/-^ mice with Ang II infusion showed reduced myocardial perivascular and interstitial fibrosis compared to WT mice with Ang II infusion. In WT mice, Ang II infusion increased reactive oxygen species formation and the expression of NADPH oxidase subunits, indicating increased oxidative stress, but not in TNF-α^-/-^ mice. In addition, treatment with etanercept (8 mg/kg, every 3 days) for two weeks blunted the Ang II-induced hypertension (133±4 vs 154±3 mmHg, p<0.05) and cardiac hypertrophy (heart weight to body weight ratio, 4.8±0.2 vs 5.6±0.3, p<0.05) in WT mice. Furthermore, Ang II-induced activation of NF-κB, p38 MAPK, and JNK were reduced in both TNF-α^-/-^ mice and mice treated with etanercept. Together, these findings indicate that TNF-α contributes to Ang II-induced hypertension and adverse cardiac remodeling, and that these effects are associated with changes in the oxidative stress dependent MAPK/TGF-β/NF-κB pathway. These results may provide new insight into the mechanisms of Ang II and TNF-α interaction.

## Introduction

Angiotensin II (Ang II) plays an important role in blood pressure regulation and cardiac hypertrophy. Multiple signaling pathways that regulate Ang II-mediated cardiac hypertrophy and hypertension have been identified; these include activation of protein kinase-C, mitogen activated protein kinases (MAPK), and the production of reactive oxygen species (ROS) and reactive nitrogen species (RNS) [1]. Ang II enhances the production of ROS through stimulation of NADPH oxidase via the type 1 receptors (AT_1_R) [2,3]. Increased oxidative stress contributes to endothelial dysfunction and vascular inflammation by stimulating redox-sensitive transcription factors such as NF-κB and up-regulating adhesion molecules, cytokines, and chemokines [4].

Tumor necrosis factor-α (TNF-α) is a proinflammatory cytokine with a wide range of biological effects and has been implicated in the pathophysiology of many cardiovascular diseases, including hypertension [5–7]. TNF-α is central in initiating and sustaining the proinflammatory cytokine cascade and can stimulate the production of other cytokines such as IL-1 and IL-6 [8]. TNF-α has been shown to increase the production of ROS in cultured cardiac myocytes [9]. Additionally, TNF-α overexpression in transgenic mice causes adverse cardiac remodeling, characterized by increased total matrix metallo protein (MMP) activity and increased fibrosis [10,11].

Both Ang II and TNF-α were shown to induce activation of NF-κB in a ROS-dependent manner, which in turn can increase the production of other proinflammatory cytokines and chemokines [4,6,7]. Several studies have established the role of ROS in hypertrophy and remodeling of the heart and blood vessels, which contribute further to the pathogenesis of hypertension [12,13]. It is well established that there is increased production of superoxide and depletion of nitric oxide (NO) in the endothelium and in the heart of hypertensive animals, and this contributes to contractile dysfunction. This depletion in nitric oxide could be either due to a direct decrease in NO production (inhibition/depletion of nitric oxide synthase [NOS]) or due to decreased bioavailability of NO because of its interaction with superoxide to form peroxynitrite [3,12]. In Ang II-infused animal models and spontaneously hypertensive rats (SHR), NADPH activity is increased and ROS generation is enhanced. These processes are mediated through AT_1_Rs and associated with overexpression of vascular and cardiac NADPH oxidase subunits [14–16]. It has been shown that Ang II-induced superoxide production and hypertension are markedly reduced in mice lacking the p47phox subunit of NADPH oxidase [17]. Treatment with the cell permeable superoxide dismutase (SOD) mimetic tempol lowers blood pressure in the 1-kidney, 1-clip model of renovascular hypertension [18], and in the SHR model of hypertension [19]. Studies using vascular smooth muscle cells, vascular fibroblasts, endothelial cells, and cardiomyocytes demonstrated mRNA and protein expression of gp91phox increased in response to Ang II stimulation [2]. These studies support a role for NADPH oxidase-derived ROS and increased oxidative stress in the pathogenesis of Ang II-induced cardiac hypertrophy and hypertension.

Blockade of TNF-α has been shown to delay the progression of salt sensitive hypertension [20], prevent the development of hypertension in fructose fed rats [21], and decreased hypertension in a mouse model of systemic lupus erythematosus [22]. Previous studies from this and other laboratories have shown that inhibition of TNF-α was involved in attenuating Ang II mediated hypertension [5,7,23,24]. However, the signaling mechanisms involved in these responses have not been elucidated. In the present study, we have examined whether TNF-α contributes to Ang II-induced hypertension and adverse cardiac remodeling through oxidative stress and NF-κB mediated signaling. We also investigated the role of TNF-α inhibition on activation of MAPK signaling in Ang II-induced hypertension and cardiac hypertrophy. To address these questions, we have used two different approaches to inhibit TNF-α: TNF-α knockout mice and pharmacological blockade of TNF-α with etanercept, a clinically available recombinant TNF-α receptor that reduces the biological activity of TNF-α. The findings from this study contribute to our understanding of the cellular signaling mechanisms of TNF-α in Ang II-induced hypertension and cardiac remodeling.

## Methods

### Ethics Statement

All experimental procedures were in compliance with all applicable principles set forth in the National Institutes of Health 2011 Guide for the Care and Use of Laboratory Animals. This study was approved by the Institutional Animal Care and Use Committee of the Louisiana State University School of Veterinary Medicine (protocol number 09–008).

### Experimental Animals

Male, 8–10 week-old, wild-type (WT) and TNF-α knockout (TNF-α^-/-^) mice were implanted with radio-telemetry transmitters to measure blood pressure monitoring as described previously [5]. A week later, baseline blood pressure was recorded for 3 days. Then mice were implanted with osmotic minipumps to receive either Ang II (1μg/kg/min) or vehicle saline for 14 days. For each surgery, mice were anesthetized with isoflurane (2%) in an oxygen flow (1 L/min) and placed on a heating pad to maintain body temperature. Post-operative care, included a buprenorphine injection to relieve pain at the end of the surgery and after 12 hours (0.05 mg/Kg, sc). Animals were housed in a temperature- and humidity-controlled facility under a 12 hour dark/light cycle, fed standard mouse chow and water *ad libitum*. The mice groups are as follows: 1) WT+Saline—control mice with saline pumps; 2) WT+Ang II—wild type treated with chronic Ang II; 3) TNF-α^-/-^+saline—knockout mice with saline pumps; 4) TNF-α^-/-^+Ang II—knockout mice treated with chronic Ang II. In another set of experiments, 8 mg/kg etanercept was administered i.p. 3 days before and every 3 days during saline or Ang II infusion for 14 days [23]. At the end of 14 days, the mice were euthanized by carbon dioxide inhalation and the hearts were collected for further analyses.

### Real Time RT-PCR

Total RNA was isolated from left ventricular tissue with RNeasy kit (Qiagen), and cDNA was synthesized from 1μg RNA with the iScrpt cDNA synthesis kit (Bio-Rad). Real Time PCR amplification reactions were performed with iQ SYBR Green Super mix with ROX (Bio-Rad) in triplicate using the ABI Prism 7900 Real time PCR machine (Applied Biosystems). The specificity of SYBR green assays were confirmed by dissociation curve analysis. Data were normalized to GAPDH expression by the ΔΔC_T_ comparative method and expressed as fold change compared to control.

### Western Blot Analysis

The left ventricular tissue was homogenized with RIPA lysis buffer and protein lysates were separated by centrifugation. The total protein concentration in samples was measured by Bio-Rad Dc protein assay. Equal amounts of protein (30 μg) were separated by SDS-PAGE on 10% gels and transferred on to PVDF membrane (Immobilon-P, Millipore; Bedford, MA), and blocked with 1% BSA in TBS-T at room temperature for 60 minutes. The membranes were subjected to immunoblot analyses with primary antibody (1:1000 dilution). Immunodetection was accomplished with a HRP conjugated secondary antibody (1:5000 dilution) using an enhanced chemiluminescence kit (Amersham). Protein expression of GAPDH was used as a loading control. The data were quantified by densitometry using NIH Image J software.

### Measurement of Collagen

To determine collagen content, heart sections (10μm thickness) were cut and stained with Picro-Sirius Red. Fibrillar collagen was identified in the Picro-Sirius stained sections by its red appearance. With the use of NIH Image J software, these sections were analyzed morphometrically. The area of perivascular fibrosis was calculated as the ratio of the fibrosis area surrounding the vessel to the total vessel area.

### Measurement of Total ROS, Superoxide and Peroxynitrite

Total ROS, superoxide and peroxynitrite were measured in left ventricular tissue using a bench top electron paramagnetic resonance (EPR) spectrometer e-scan R (Noxygen science transfer and diagnostics, Elzach, Germany), as previously described [25–27].

### Measurement of NF-κB Activity

Hearts were harvested from the mice and nuclear extracts were obtained using a commercially available nuclear extraction kit (Active Motif, Carlsbad, CA). Protein concentrations were then quantified using a Bio-Rad protein assay. Equal amounts of protein were utilized in a colorimetric NF-κB assay specific for the activated form of the p65 subunit of NF-κB using a commercially available kit (TransAm NF-κB p65, Active Motif).

### Statistical Analysis

Data are presented as mean ± SEM. Data were analyzed, when appropriate, by Student’s *t* test, repeated measures ANOVA, or 1-way ANOVA followed by a post-hoc Bonferroni’s test for multiple comparisons between means. Statistical comparisons were performed using Prism5 (GraphPad Software). Values of *p*<0.05 were considered statistically significant.

## Results

### Profibrotic Gene Expression and Cardiac Fibrosis

**Fig 1:**
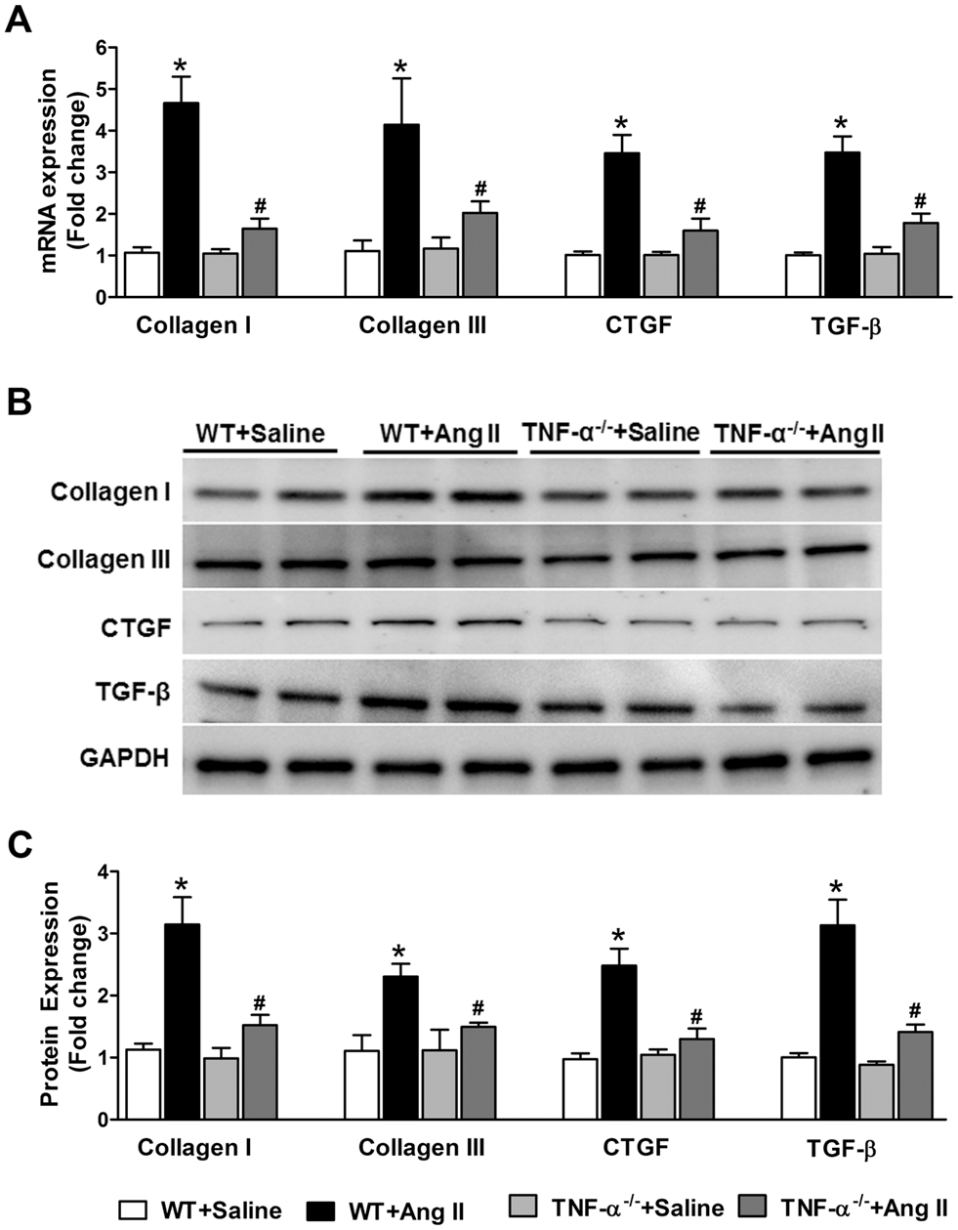
Effect of Ang II on mRNA and protein expression of profibrotic genes in WT and TNF-α^-/-^ mice. Ang II infusion significantly increased mRNA and protein expression of collagen I, collagen III, CTGF, and TGF-β in the hearts of WT mice, which was prevented in TNF-α^-/-^ mice. (a) mRNA expression of profibrotic genes in the heart. (b) Representative western blot images for cardiac collagen I, collagen III, CTGF, and TGF-β protein expression in WT and TNF-α^-/-^ mice with saline or Ang II infusion for 14 days. (c) Densitometric quantification of western blot results. Values are mean ± SEM. *p< 0.05 vs WT+Saline, # *p*< 0.05 vs WT+Ang II, n = 6–8 per group.

**Fig 2:**
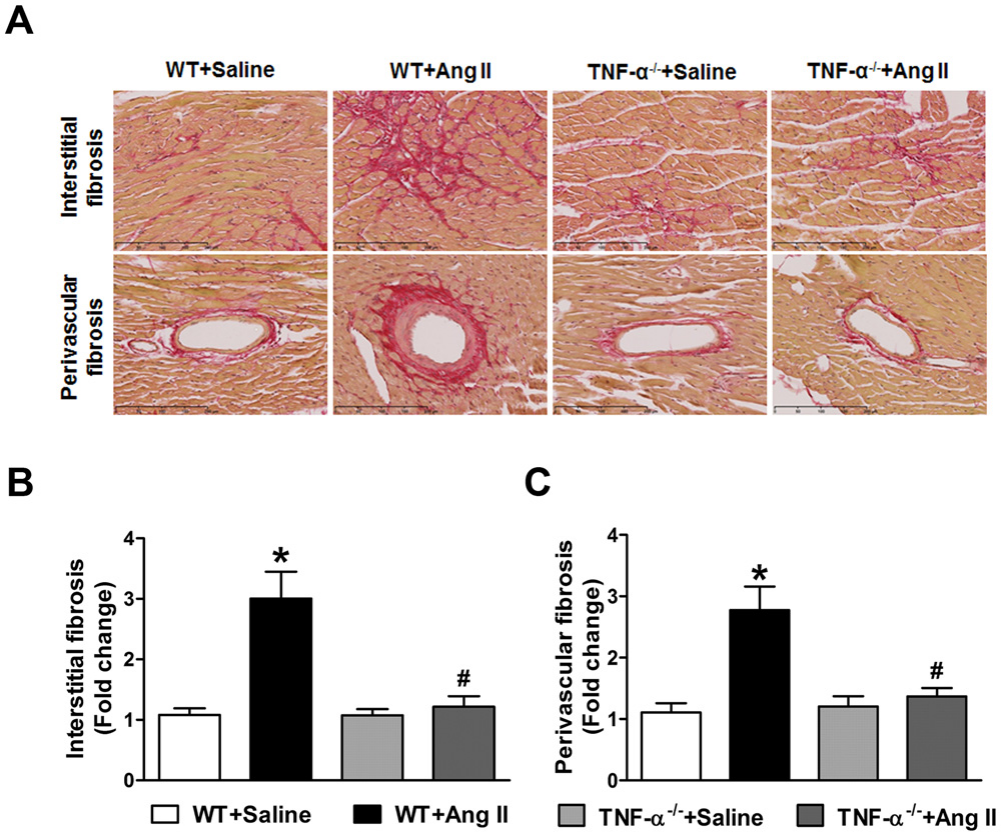
Effect of Ang II on fibrosis in WT and TNF-α^-/-^ mice. (a) The heart sections were stained with Picro-Sirius Red. Representative images are shown. (b) and (c) Densitometric quantification Values are mean ± SEM. *p< 0.05 vs WT+Saline, # *p*< 0.05 vs WT+Ang II, n = 6 per group.

Ang II-induced hypertension is characterized by increased profibrotic gene expression and cardiac fibrosis. To determine whether profibrotic gene expression in the heart was altered in TNF-α^-/-^ mice, we analyzed cardiac expression of collagen I, collagen III, connective tissue growth factor (CTGF), and TGF-β which were shown to be involved in cardiac fibrosis [28]. Ang II infusion significantly increased both mRNA and protein expression of collagen I, collagen II, CTGF and TGF-β in WT mice hearts compared with saline treated mice. This increase in Ang II-induced profibrotic gene and protein expression was markedly attenuated in TNF-α^-/-^ mice (Fig 1). To further investigate the role of TNF-α blockade in the regulation of end organ damage following Ang II-infusion, we examined the collagen deposition in the interstitial space and coronary arteries of heart sections by Picro-Sirius Red staining (Fig 2). Chronic Ang II infusion for 14 days showed a significant increase in interstitial fibrosis and perivascular fibrosis as indicated by increased collagen deposition in the interstitial and perivascular space of coronary arteries in the hearts of WT mice. In contrast, no significant change in interstitial and perivascular fibrosis was observed in Ang II infused TNF-α^-/-^ mice (Fig 2). Taken together, these data suggest that TNF-α inhibition attenuated cardiac fibrosis in response to Ang II infusion, and that the cardiac fibrosis response of Ang II is, in part, contributed by TNF-α. Therefore, blocking TNF-α is beneficial in preventing the cardiac fibrosis and remodeling induced by AngII-induced hypertension.

### Cardiac Oxidative Stress

**Fig 3:**
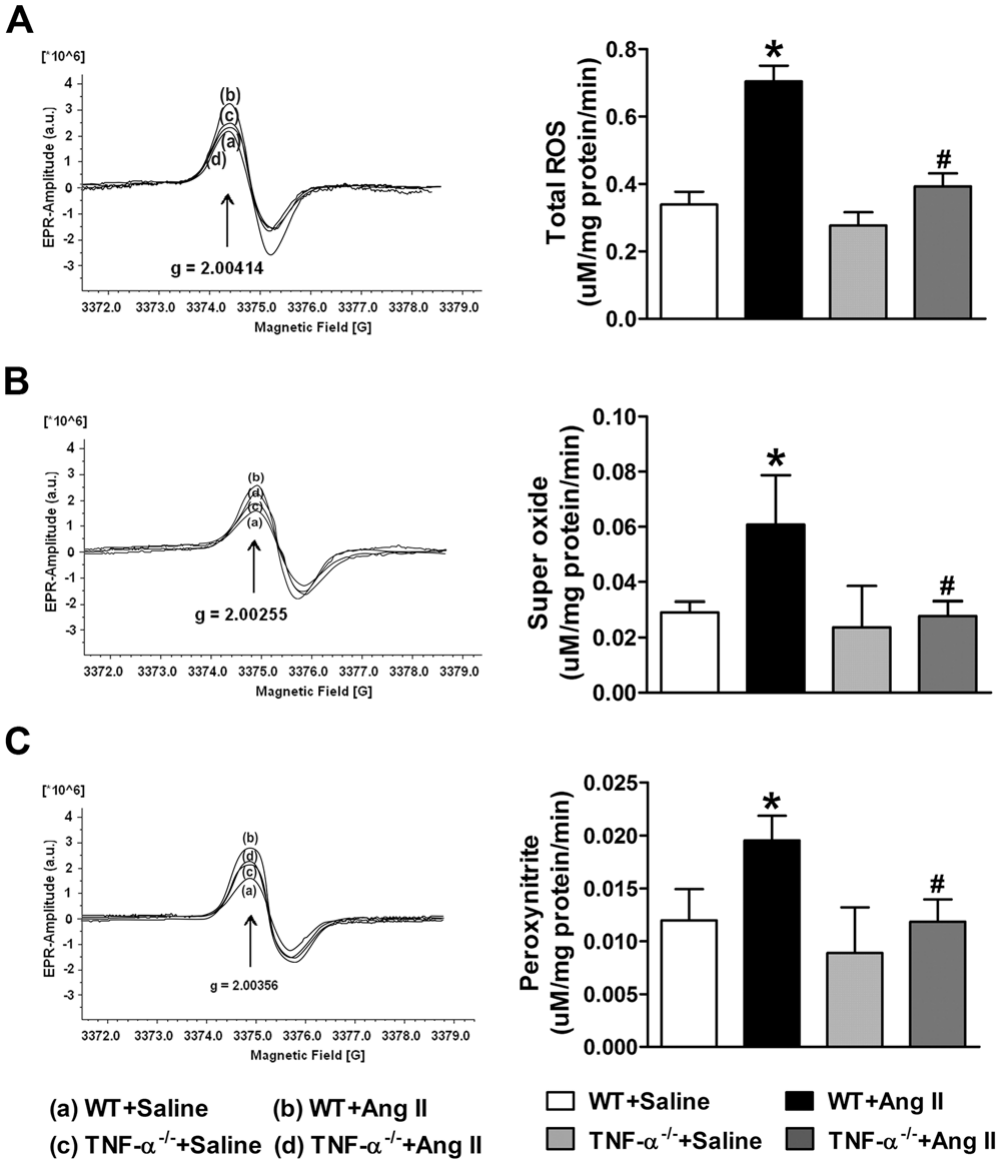
Effect of Ang II cardiac oxidative stress in WT and TNF-α^-/-^ mice. (a) Total ROS, (b) superoxide, (c) peroxynitrite production rates in cardiac tissue are measured by electron paramagnetic resonance spectroscopy. Ang II infusion increased total ROS, superoxide and peroxynitrite production in the hearts of WT mice but not in TNF-α^-/-^ mice. Values are mean ± SEM. *p< 0.05 vs WT+Saline, # *p*< 0.05 vs WT+Ang II, n = 6–8 per group.

ROS have been shown to act as important signaling molecules in the cardiovascular system and activate many signaling pathways mediated by Ang II. To evaluate the effect of Ang II on ROS formation in the left ventricle, we measured and quantified total ROS, superoxide and peroxynitrite production in the heart tissue by electron paramagnetic resonance (Fig 3). Ang II infusion significantly increased the total ROS, superoxide and peroxynitrite production in WT mice compared with saline infused control mice, leading to increased oxidative stress. These changes were attenuated in TNF-α^-/-^ mice infused with Ang II.

### Activation of NADPH Oxidase

**Fig 4:**
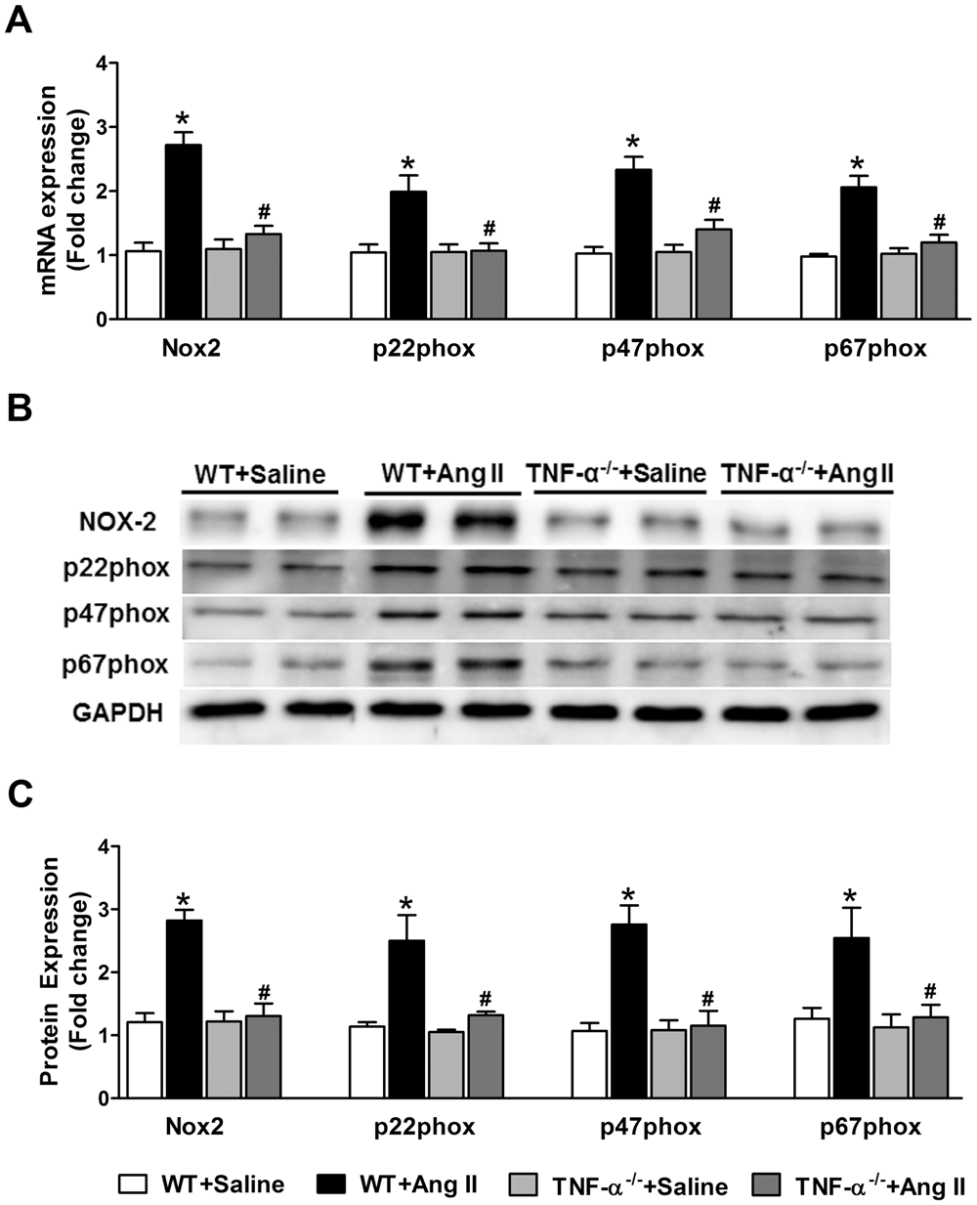
Effect of Ang II on mRNA and protein expression of NADPH oxidase subunits in WT and TNF-α^-/-^ mice. (a) Ang II infusion increased NOX-2, p22phox, p47phox and p67phox mRNA expression in the hearts of WT mice but not in TNF-α^-/-^ mice. (b) Representative western blots showing protein levels of the NADPH subunits in heart tissue. (c) Densitometric quantification of western blots. Values are mean ± SEM. *p< 0.05 vs WT+Saline, #*p*< 0.05 vs WT+Ang II, n = 6–8 per group.

Because Ang II induced oxidative stress through stimulation of NADPH oxidase, we investigated the cardiac expression of the NADPH oxidase subunits Nox2, p22phox, p47phox and p67phox. We observed that TNF-α^-/-^ mice had no changes in myocardial expression of NADPH oxidase enzyme subunits when compared with WT control mice (Fig 4a). Chronic Ang II infusion caused a significant increase in the mRNA expression of NADPH oxidase subunits in WT mice. However, these changes in mRNA were attenuated in Ang II infused TNF-α^-/-^ mice (Fig 4a). Also, these mRNA changes were confirmed with protein expression by western blot analysis, which showed similar results (Fig 4b and 4c).

### Activation of NF-κB by Ang II-infusion

**Fig 5:**
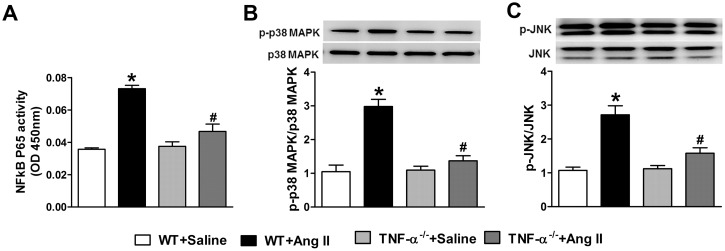
Effect of Ang II on NF-κB activity and phosphorylation of p38 MAPK and JNK, in WT and TNF-α^-/-^ mice. (a) Ang II infusion increased cardiac NF-κB p65 activity in WT mice but not in TNF-α^-/-^ mice. Phosphorylation of p38 MAPK (b) and JNK (c) was significantly increased by Ang II infusion for 14 days in WT mice, while TNF-α^-/-^ mice showed no change. Values are mean ± SEM. *p< 0.05 vs WT+Saline, # *p*< 0.05 vs WT+Ang II, n = 6 per group.

Both Ang II and TNF-α can induce oxidative stress, which in turn, activates transcription factors such as NF-κB. In our study, the activity of p65 NF-κB in nuclear extracts of the cardiac tissue was significantly increased in Ang II infused WT mice compared to saline infused WT mice (Fig 5a). However, Ang II infused TNF-α^-/-^ mice showed a significantly decreased activity of p65 NF-κB in the heart compared to WT mice following Ang II infusion. These results suggest that Ang II-induced cardiac NF-κB activity requires TNF-α.

### MAPK signaling mechanisms

To better understand the signaling mechanisms involved in attenuation of Ang II-induced effects by TNF-α inhibition, we investigated the phosphorylation of p38 MAPK and JNK in cardiac tissue of these mice. As shown in Fig 5b and 5c, protein levels of p38 MAPK and JNK were unchanged in response to Ang II infusion, but their phosphorylation increased significantly during Ang II-infusion in WT mice. Ang II-infused TNF-α^-/-^ mice had reduced phosphorylation of p38 MAPK and JNK, suggesting that TNF-α is important for signaling.

### Chronic TNF-α blockade with Etanercept

**Fig 6:**
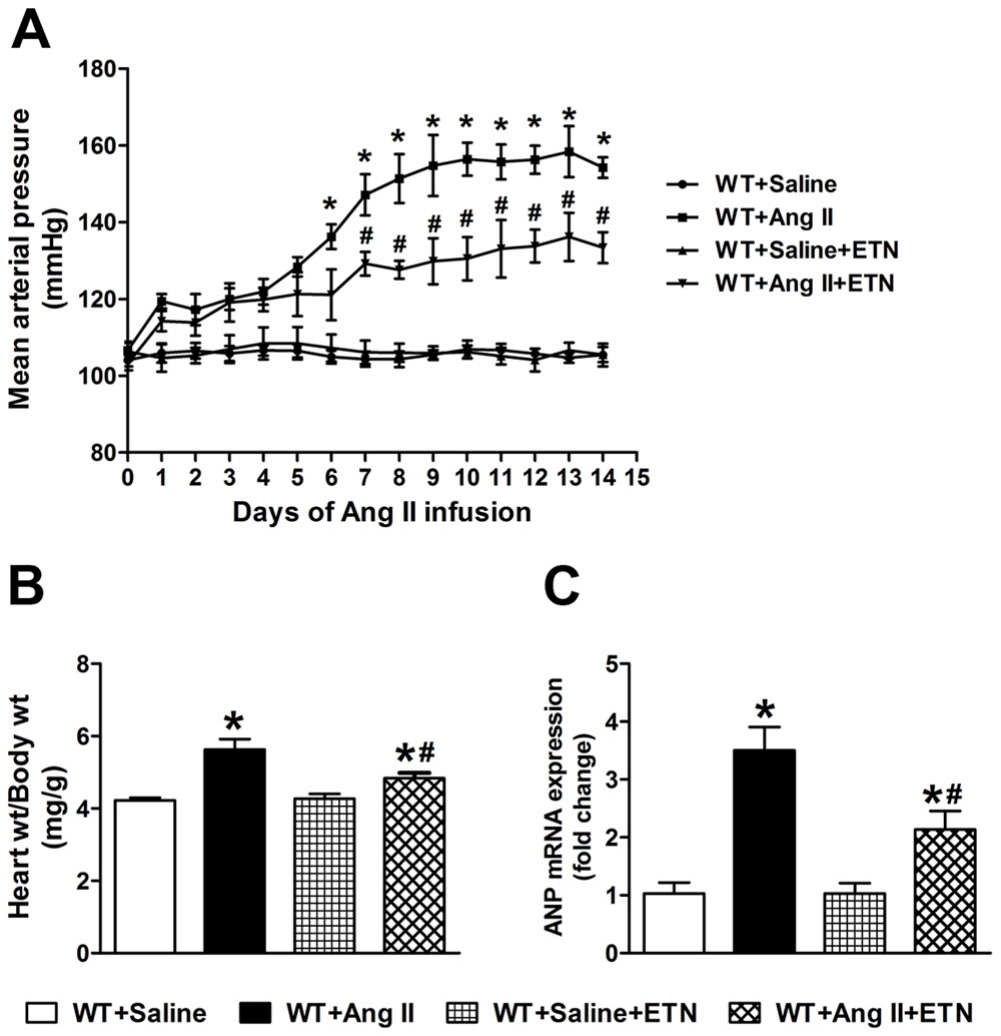
Effect of TNF-α inhibition with etanercept on Ang II-induced hypertension and cardiac hypertrophy. (a) Chronic Ang II infusion for 14 days significantly increased mean arterial pressure in WT mice, which was attenuated by treatment with etanercept. (b) Ang II induced cardiac hypertrophy, as assessed by heart weight to body weight ratio, was attenuated by etanercept treatment. (c) Cardiac hypertrophy marker ANP gene expression in the hearts of Ang II-infused mice was significantly increased. Etanercept treatment for 14 days attenuated this Ang II-induced increase in ANP expression. Values are mean ± SEM. *p< 0.05 vs WT+Saline, # *P*< 0.05 vs WT+Ang II, n = 6 per group.

**Fig 7:**
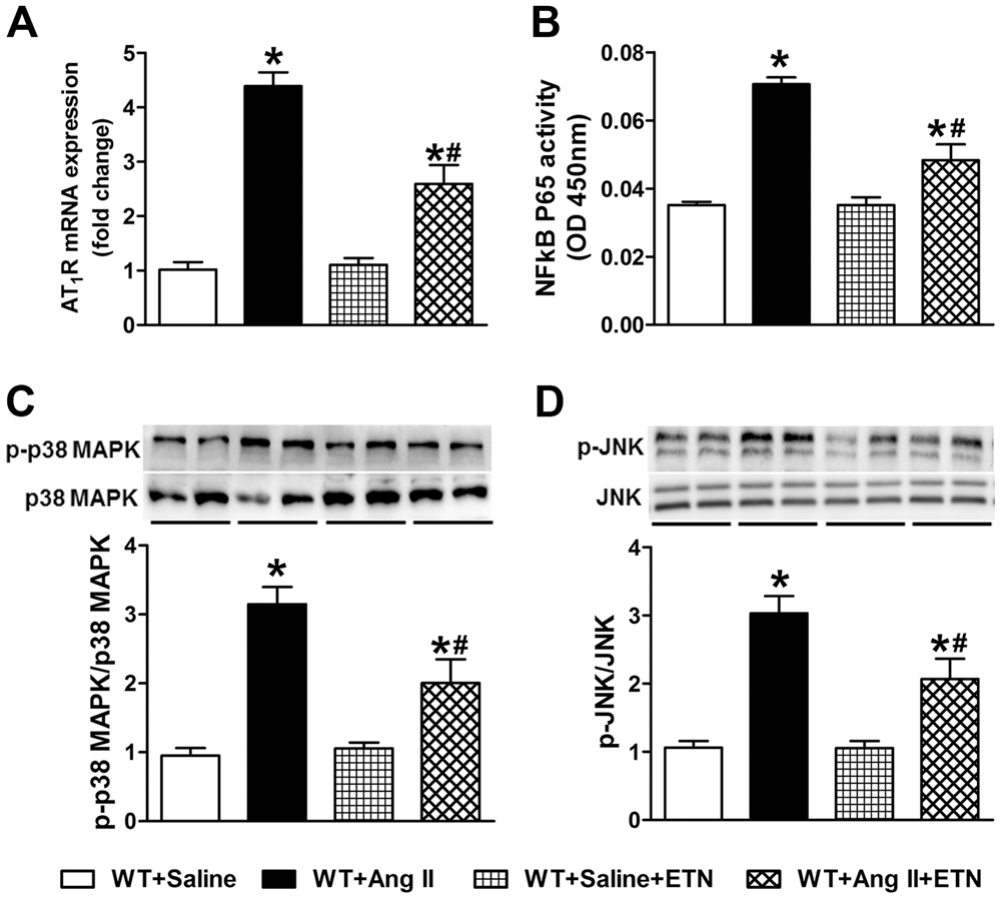
Effect of TNF-α inhibition with etanercept on Ang II-induced AT_1_R gene expression, NF-κB activity, and phosphorylation of p38 MAPK and JNK in WT mice. (a) AT_1_R gene expression was significantly elevated in heart tissue by Ang II infusion, which was attenuated by etanercept treatment. (b) Chronic Ang II infusion significantly increased cardiac NF-κB p65 activity in WT mice but not in mice treated with etanercept. (c) p38 MAPK and (d) JNK phosphorylation in the hearts of Ang II-infused mice was significantly increased. Etanercept treatment prevented this Ang II-induced phosphorylation. Values are mean ± SEM. *p< 0.05 vs WT+Saline, # *p*< 0.05 vs WT+Ang II, n = 6 per group.

In order to further confirm the role of TNF-α in Ang II-induced hypertension and cardiac hypertrophy, mice were treated with etanercept during saline or Ang II infusion for 14 days. Etanercept treatment alone had no significant effect on blood pressure (Fig 6a). Chronic Ang II infusion significantly increased mean arterial pressure and cardiac hypertrophy in WT mice, while, TNF-α inhibition with etanercept resulted in attenuated Ang II-induced hypertensive (Fig 6a) and cardiac hypertrophic responses (Fig 6b). Furthermore, cardiac gene expression analysis of hypertrophy marker ANP (Fig 6c) showed reduced expression in Ang II-infused mice treated with etanercept suggesting an attenuated cardiac hypertrophy. Etanercept treatment also attenuated Ang II-induced increases in AT_1_R expression (Fig 7a), NF-κB activity (Fig 7b), and phosphorylation of p38 MAPK and JNK in cardiac tissue (Fig 7c and 7d). These results further confirm that TNF-α inhibition attenuates Ang II-induced hypertensive and cardiac hypertrophic response by preventing the phosphorylation of p38 MAPK and JNK.

## Discussion

Several studies have suggested that a functionally significant cross-talk exists between Ang II and inflammatory cytokines such as TNF-α, which may participate in self-sustaining and/or self-amplifying positive feedback loops in the development of hypertension and cardiac remodeling [29–31]. Previously, using TNF-α gene deficient mice, we have shown that inhibition of TNF-α attenuates an Ang II-induced hypertensive response [5]. In this study, using a genetic knockout animal model and pharmacological blockade of TNF-α, we show for the first time that attenuation of Ang II mediated hypertension and cardiac hypertrophy by TNF-α inhibition involves decreased oxidative stress and NF-κB activation, and reduced phosphorylation of MAPK.

TNF-α induces myocardial fibrosis by inhibiting collagen phagocytosis in the heart [32] and up-regulating AT_1_R on cardiac fibroblasts, leading to enhancement of the effects of Ang II [33]. Transgenic mice with cardiac restricted overexpression of TNF-α develop progressive myocardial fibrosis, possibly due to TNF-α mediated signaling of AT_1_R [10,11,34]. In our study, a marked attenuation of Ang II-induced cardiac hypertrophy and fibrosis was demonstrated in TNF-α^-/-^ mice, as indicated by decreased profibrotic gene expression and decreased interstitial and perivascular fibrosis in the cardiac tissue. A study by Sun et al., showed that locally generated TNF-α is involved in cardiac remodeling by stimulating cardiac hypertrophy and fibrogenic response in a pressure overloaded heart through modulation of CTGF and TGF-β expression, ultimately leading to ventricular dysfunction [7]. CTGF is well known for its role as a downstream mediator of the chronic fibrotic effects of TGF-β, which, when activated stimulates fibroblasts to differentiate into myofibroblasts, the key cells for collagen synthesis in cardiac remodeling [28,35]. In our study, we found enhanced CTGF and TGF-β expression in Ang II-infused WT mice but not in TNF-α^-/-^ mice. These results complement the previous studies and suggest that the effects of Ang II on cardiac hypertrophy and fibrosis are mediated by TNF-α mediated enhanced myocardial CTGF and TGF-β expression.

Oxidative stress and increased ROS have been implicated in hypertension in human studies of essential hypertension and several experimental models of hypertension [3,36,37]. Many studies have shown that Ang II enhances ROS generation by activating NADPH oxidase via AT_1_R activation, which in turn activates redox sensitive transcription factors such as NF-κB [12]. Ang II-mediated superoxide generation requires the functionally active NADPH subunits Nox-2, p22phox, p47phox, and p67phox; downregulation or absence of these subunits results in attenuated or abrogated Ang II-induced cell growth, contraction, and inflammation [13,38], whereas upregulation is associated with enhanced effects [39]. Additionally, TNF-α can induce oxidative stress by activating NADPH oxidase and decreasing the release of NO [6,40,41]. We found that TNF-α^-/-^ mice infused with Ang II did not show any significant changes in total ROS generation, superoxide and peroxynitrite production, and NADPH subunit activation, suggesting an attenuated oxidative response. This result is in agreement with previous studies showing that TNF-α is involved in ROS generation, and blockade of TNF-α can decrease the production of ROS and attenuate the Ang II induced hypertension [23].

Another major finding in the current study was the identification of the central role of TNF-α in the effect of p38 MAPK and JNK signaling on Ang II-induced hypertension and cardiac hypertrophy. Both Ang II and TNF-α can activate p38 MAPK and JNK signaling pathways in cardiac myocytes [29]. MAPK are important regulators of the effects of Ang II on tissue structure, which is, at least in part, responsible for Ang II-induced TGF-β which in turn up regulates CTGF leading to cardiac remodeling [42]. We measured cardiac phosphorylation of p38 MAPK and JNK and found increased phosphorylation of p38 MAPK and JNK in Ang II infused WT mice. This activation was blunted in TNF-α^-/-^ mice infused with Ang II. Recent evidence suggests that one of the most important downstream signaling molecules common for both TNF-α and Ang II is the transcription factor NF-κB. NF-κB is not only involved in the activation of proinflammatory cytokines, but also in the induction of oxidative and nitrosative stress [4]. Moreover, pathophysiologically relevant concentrations of Ang II are sufficient to provoke TNF-α mRNA and protein synthesis in the adult heart through a NF-κB dependent pathway [43]. Our lab has shown that increases in Ang II-mediated NF-κB p50 mRNA expression were attenuated in TNF-α^-/-^ mice with chronic Ang II-infusion [5]. In the present study, we found that TNF-α^-/-^ mice infused with Ang II showed attenuated NF-κB p65 activity in the cardiac tissue. Overall, these results suggest that the beneficial effects of TNF-α inhibition are, at least in part, mediated by the MAPK/TGF-β/NF-κB pathway.

We further confirmed our transgenic animal study results with pharmacological inhibition of TNF-α by etanercept, a clinically available recombinant TNF-α receptor that reduces the biological activity of TNF-α. Treatment with etanercept has been shown to delay the progression of salt sensitive hypertension [20], prevent the development of hypertension in fructose fed rats [21], and decrease hypertension in a mouse model of systemic lupus erythematosus [22]. Etanercept treatment also prevented the hypertension and increase in vascular superoxide and oxidative stress induced by Ang II treatment [23,24]. Our study extends these findings and showed that treatment with etanercept prevented the Ang II-induced hypertension and cardiac hypertrophy mediated by decreased activation of p38 MAPK and JNK.

In summary, our studies further elucidate the mechanisms for the TNF-α signaling pathway in Ang II-induced hypertensive and hypertrophic response and demonstrate the novel findings that TNF-α is involved in Ang II-mediated cell signaling, which leads to cardiac hypertrophy, fibrosis and the hypertensive response. Taken together, these data suggest that Ang II-induced cardiac hypertrophy and hypertension are dependent on the presence of NADPH oxidase, increased oxidative stress, and activation of NF-κB, and require concomitant generation of TNF-α. More importantly, this study shows that the attenuation of the Ang II-induced hypertensive and hypertrophic response by blockade of TNF-α is mediated by decreased oxidative stress (decreased total ROS, superoxide, and peroxynitrite) and downregulation of the MAPK/TGF-β/NF-κB pathway.
